# Diet intervention improves cardiovascular profile in patients with rheumatoid arthritis: results from the randomized controlled cross-over trial ADIRA

**DOI:** 10.1186/s12937-021-00663-y

**Published:** 2021-01-23

**Authors:** Erik Hulander, Linnea Bärebring, Anna Turesson Wadell, Inger Gjertsson, Philip C. Calder, Anna Winkvist, Helen M. Lindqvist

**Affiliations:** 1 Department of Internal Medicine and Clinical Nutrition, Institute of Medicine, Sahlgrenska Academy, University of Gothenburg, PO Box 459, SE-405 30 Gothenburg, Sweden; 2 Department of Rheumatology and Inflammation Research, Institute of Medicine, Sahlgrenska Academy, University of Gothenburg, Gothenburg, Sweden; 3 Faculty of Medicine, University of Southampton and NIHR Southampton Biomedical Research Centre, University Hospital Southampton NHS Foundation Trust, Tremona Road, Southampton, SO16-6YD UK

**Keywords:** Rheumatoid Arthritis, Diet Therapy, Cardiovascular Diseases, Cross-Over Studies, Lipoproteins, Apolipoproteins B

## Abstract

**Background:**

The chronic inflammation in patients with rheumatoid arthritis (RA) increases the risk for cardiovascular diseases (CVD). The contribution of diet as a risk factor for CVD among these patients is however not fully understood. The aim of this study is to investigate if a proposed anti-inflammatory diet improves cardiovascular profile in weight stable patients with RA.

**Methods:**

Patients (*n* = 50) with RA were included in a cross-over trial. They were randomized to either a diet rich in whole grain, fatty fish, nuts, vegetables and fruit and supplemented with probiotics, or a control diet resembling average nutritional intake in Sweden, for ten weeks. After a 4-month washout they switched diet. Participants received food bags and dietary guidelines. Primary outcome was triglyceride (TG) concentration. Secondary outcomes were total-, high density lipoprotein- (HDL) and low density lipoprotein- (LDL) cholesterol, Apolipoprotein-B100 and -A1, lipoprotein composition, plasma phospholipid fatty acids and blood pressure.

**Results:**

Forty-seven patients completed at least one period and they remained weight stable. There was a significant between-dietary treatment effect in TG and HDL-cholesterol concentration in favor of intervention (*p* = 0.007 and *p* = 0.049, respectively). Likewise, Apolipoprotein-B100/A1 ratio shifted toward a less atherogenic profile in favor of the intervention (*p* = 0.007). Plasma fatty acids increased in polyunsaturated- and decreased in monounsaturated- and saturated fatty acids between diet periods in favor of the intervention period.

**Conclusion:**

Blood lipid profile improved indicating cardioprotective effects from an anti-inflammatory dietary intervention in patients with RA.

**Trial registration:**

This trial is registered at ClinicalTrials.gov as NCT02941055.

**Supplementary Information:**

The online version contains supplementary material available at 10.1186/s12937-021-00663-y.

## Introduction

Rheumatoid arthritis (RA) is a chronic autoimmune disease that is characterized by systemic inflammation and joint damage. Patients with RA have a shorter life expectancy compared to the general population [1], and have a substantially increased risk of cardiovascular diseases (CVD) [2]. Even though pharmacological treatment has improved in recent decades, risk of CVD compared to the general population remains elevated [3].

For the general population, primary risk factors for CVD include dyslipidemia, e.g. elevated low density lipoprotein (LDL) cholesterol and triglyceride (TG), low high density lipoprotein (HDL) cholesterol, as well as high Apolipoprotein-B100 (Apo-B100) concentrations [4] .In RA, studies investigating dyslipidemia in relation to CVD-events have not always shown consistent results. Some reports have found an inverse association, where lower LDL-concentration correlates to an increased risk of CVD [5], commonly referred to as the lipid-paradox. It has been demonstrated that ongoing inflammation causes an increased clearance of LDL-cholesterol from the circulation, but not necessarily a decreased risk of CVD [6]. Also, an increase in remnants of the triglyceride transporting protein Apolipoprotein B-48 in patients with RA compared to others with high risk of CVD has been seen [7], suggesting an altered blood lipid metabolism. An increased disease activity and inflammation in RA is associated with an altered blood lipid profile and anti-inflammatory pharmacological therapy reduces these alterations [6]. Mediterranean-like diets have shown promising effects on improving CVD health in the general population [8]. To what extent a dietary manipulation can improve blood lipid profile and reverse CVD risk profile in patients with RA remains to be clarified.

### Aim

The aim of this study was to evaluate the effect of a proposed anti-inflammatory diet during stable disease activity in patients with RA on cardiovascular risk factors.

## Methods

### Ethics

This study was approved by the regional ethical review board in Gothenburg (976–16 and T519–17) and registered on ClinicalTrials.gov (NCT02941055). All procedures were conducted according to the Declaration of Helsinki. All participants provided signed informed consent prior to participation.

### Recruitment

Eligible patients with RA according to ACR 1987 and ACR/EULAR 2010 criteria [9] at the Sahlgrenska University Hospital, Gothenburg, Sweden, were identified through the Swedish Rheumatology Quality Register. Those who resided in areas where home food delivery was possible were sent study invitation by post.

Inclusion criteria were active disease defined as DAS28-ESR ≥2.6, stable disease activity defined as no changes in disease-modifying antirheumatic drugs (DMARD) therapy during the past 8 weeks, age 18–75 years, and at least 2 years disease duration. Exclusion criteria were other life threatening diseases, pregnancy, lactation, intolerance or allergy to foods included in the trial, or inability to communicate in Swedish.

### Study design

To minimize effects of inter-individual variance, a cross-over design was chosen. Patients were randomly assigned to begin with either intervention or control diet. A computer generated list was used to randomize participants; sequence of each patient was revealed to the study team at the baseline visit. At the start of each diet period participants received dietary instructions, and during the diet periods, food bags were delivered on a weekly basis. Each diet period lasted ten weeks with a 4-month wash-out period in between. For practical reasons the study ran in two batches, where the first group of participants entered the study in February 2017 and the second in September 2017.

### Intervention diets

The dietary interventions have been described elsewhere [10, 11]. In brief, participants received home delivery of food items equivalent to approximately half the daily intake for 5 days per week during both diet periods (Table 1), and the dietary instructions were in line with the type of foods delivered. The diet periods were designed to be isocaloric and participants were encouraged to keep a stable weight throughout the study.

**Table 1:** Daily nutritional content of food items delivered to participants

	Intervention period	Control period
Energy (kcal)	1100	1100
Protein (g)	45.8	62.3
Carbohydrate (g)	119	129
Fat (g)	43.5	34.5
Saturated fatty acids (g)	11.8	16.4
Monounsaturated fatty acids (g)	14.3	11.4
Polyunsaturated fatty acids (g)	13.5	3.84
n-6 fatty acids (g)	9.53	3.03
n-3 fatty acids (g)	3.51	0.78
Σ DHA, DPA & EPA (g)	2.27	0.04
Cholesterol (mg)	86.3	139
Fiber (g)	23.9	8.26
Starch (g)	11.8	46.7
Whole grain products (g)	29.8	0.79
Vitamin D (μg)	9.39	1.33

*DHA* Docosahexaenoic acid, *DPA* Docosapentaenoic acid, *EPA* Eicosapentaenoic acid

The intervention breakfast consisted of a fruit drink enriched with 2 × 10^0^ colony forming units of *Lactobacillus plantarum* 299v, frozen berries and either 1) low fat sour milk with nuts and whole grain muesli or 2) fiber-enriched oat porridge with low fat milk and walnuts or 3) low fat yoghurt and whole grain muesli. For the main meal, fish was served on average 3.8 times weekly and legume-based vegetarian meals 1.2 times per week. Vegetables and whole grains or potatoes were provided with these meals. Two fruits per day were supplied as snacks. Participants were also advised to limit intake of red meat to ≤3 times per week, keep intake of fruits, berries and vegetables to ≥5 portions per day, choose low fat dairy products, use margarine or vegetable oils for cooking and to choose whole grain products.

The control diet was designed to nutritionally reflect the average dietary intake in Sweden; breakfasts consisted of orange juice and either a mix of yoghurt and quark with corn flakes or white bread with butter and cheese. For the main meal, red meat was served on average 3.5 times per week and chicken 1.5 times per week. The meals included a smaller portion of vegetables and refined grains or potatoes. A daily serving of either quark, a protein bar or protein pudding was supplied as snacks. Participants were instructed to eat red meat ≥5 times per week, fish ≤1 times per week, limit intake of fruits, berries and vegetables to ≤5 portions per day, to choose whole fat dairy products, use butter for cooking and to avoid probiotic products.

In an attempt to blind participants, staff referred to control diet as “protein diet” and intervention diet as “fiber diet” in all communications.

A registered dietitian interviewed participants by telephone mid-period to assess compliance. Participants were here asked to rate their consumption from none (equal to 0 points), part of (1 point), or the whole menu item (2 points) for each received meal during the preceding week. Hence, for 5 days the maximum compliance score was 30 and participants scoring at least 80% (24 points) were regarded as compliant.

### Data collection

A Food Frequency Questionnaire was used to estimate dietary intake at screening, and a dietary quality index as previously described by the Swedish National Food Agency was used to score participants [12]. Participants also filled out a lifestyle questionnaire including the parental origin and educational level. Educational level was categorized into five levels, 1) junior high school, 2) 2 year senior high school, 3) ≥3 year senior high school, 4) college or university education and 5) no education. Before and after each dietary period, blood pressure was measured and fasting whole blood samples were collected by venipuncture. Serum and plasma were separated and frozen in − 80 °C until analysis.

### Laboratory analyses

Serum total-, HDL-, LDL-cholesterol and TG were analyzed at the Sahlgrenska University Hospital, Gothenburg, Sweden, by enzymatic colorimetry using a Cobas 8000 instrument from Roche Diagnostica, Scandinavia AB.

Serum Apo-B100 and Apolipoprotein A1 (Apo-A1) as well as lipoprotein concentrations were quantified by Nuclear Magnetic Resonance (NMR)-analysis; serum samples were prepared according to In Vitro Diagnostics Research (IVDr) standard operating procedures (Bruker BioSpin; www.bruker.com/products/mr/nmr/avance-ivdr.html). In brief, serum samples were thawed at room temperature for 30 min, then centrifuged at 3500 x g for 1 min at 4 °C. Thereafter, 325 μl of serum was transferred with a SamplePro L liquid handler (Bruker BioSpin) to a deepwell plate (Porvair, cat. no 53.219030) containing 325 μl NMR buffer ((75 mM sodium phosphate, pH 7.4, 0.08% 3-(trimethylsilyl)propionic-2,2,3,3-d_4_), 0.04% sodium azide, 20% v/v D_2_O) per well. The plate was shaken at 400 rotations per minute, 12 °C for 5 min in a Thermomixer Comfort (Eppendorf). Finally, 600 μl sample was transferred to 5 mm SampleJet NMR tubes with the SamplePro L. The sample tubes, deepwell plate and SampleJet rack were kept at 2 °C during the preparation in the SamplePro L robot.H NMR data was acquired on a Bruker 600 MHz Avance III spectrometer equipped with a room temperature 5 mm BBI probe and a cooled SampleJet sample changer. In brief, 1D NOESY (‘noesygppr1d’ pulse sequence), 1D CPMG (‘cpmgpr1d’) and 2D J-resolved (‘jresgpprqf’) spectra were acquired according to the standard IVDr parameter settings at 37 °C. A pre-acquisition temperature stabilization time of 300 s was used. Before measurement, all samples were kept at 6 °C in the SampleJet. Experimental parameters are available upon request. The 1D NOESY data was submitted for B.I.-Lisa lipoprotein profiling and B.I.Quant-PS 2.0.0 automatic quantification of a subset of metabolites through a remote secure Bruker server, generating in total 112 B.I.Lisa and 41 B.I.Quant-PS variables.

The data from the NMR-analysis also included an estimate of outcomes already analyzed at the Clinical Laboratory of Sahlgrenska University Hospital, Gothenburg, (TG as well as total, LDL- and HDL-cholesterol). Participants whose NMR results differed substantially from results of the standard clinical analysis were removed from the NMR-dataset (*n* = 3).

Fatty acid analysis in plasma phospholipids was performed by gas chromatography as previously described elsewhere [13]. Blood samples from EDTA tubes were centrifuged (913 *g* for 10 min) and plasma stored in − 80 °C until analysis. In short, total lipid was extracted into chloroform:methanol. Phosphatidylcholine, the major phospholipid in plasma, was isolated by solid phase extraction. Fatty acid methyl esters were formed from the extracts by heating with methanolic sulphuric acid. Fatty acid methyl esters were separated by gas chromatography on a Hewlett Packard 6890 gas chromatograph fitted with a BPX-70 column using the settings and run conditions described elsewhere [13]. Fatty acid methyl esters were identified by comparison with run times of authentic standards. Data are expressed as weight % of total fatty acids.

### Statistical analysis

The main analyses were performed by linear mixed ANCOVA model. Fixed variables were dietary treatment (intervention or control), period (the first or second diet period), sequence (beginning with intervention or control) as well as the baseline value for each outcome variable. Individual participant was included as random effect.

In order to test for confounders, an a priori set of variables were considered as covariates: age, sex, body mass index (BMI) at baseline, nicotine use (yes/no), dietary quality (index between 0 to 12) at baseline and educational level. Any covariate exerting a change in effect estimate of at least 10% was included in the analysis as a confounder. The only confounder noted was sex for analysis of HDL-3 cholesterol, and sex was therefore incorporated as a confounder in all analyses of lipids. Residuals from the outcome variables were reviewed in regards to model assumptions, and all were normally distributed.

As a sensitivity analysis, participants who fulfilled an a priori set of requirements were analyzed. In this analysis, only those who completed the whole trial, had good compliance to the diets (> 80%), and who did not discontinue or start any new DMARD-treatment, statins or glucocorticoids were included.

The potential interaction of baseline variables with treatment outcome was also examined for total-, LDL, and HDL-cholesterol and TG. The interaction variables tested included BMI, the latest Swedish version of the systematic coronary risk evaluation (SCORE 2015) [14], dietary quality, dietary fiber intake and quality of dietary fat intake. The Swedish version of SCORE 2015 contains age range from 40 to 65 years; thus participant scores were truncated to the closest risk estimate. Interaction was tested with dichotomized variables, grouped as above and below median, except for BMI where a cutoff between 18.5–25 kg/m^2^ was used for normal weight and > 25 kg/m^2^ for overweight. If an interaction was identified (defined as *p* <  0.200 for the interaction term), subgroup analyses were performed.

### Power calculation

The power analysis of the ADIRA study was performed on the primary outcome 28-joint Disease Activity Score Erythrocyte Sedimentation Rate (DAS28-ESR), previously published elsewhere [11]. In order to detect a change of 0.6 units in DAS28-ESR with 90% power and α = 0.05, a sample size of 38 patients was needed and to account for dropouts 50 patients were recruited. Primary outcome in this report is TG concentration. Secondary outcomes are total-, HDL-, LDL-cholesterol and Apo-B100 and Apo-A1 concentration, lipoprotein composition and lipoprotein particle counts, plasma phospholipid fatty acids and blood pressure.

## Results

### Subjects

**Fig. 1:**
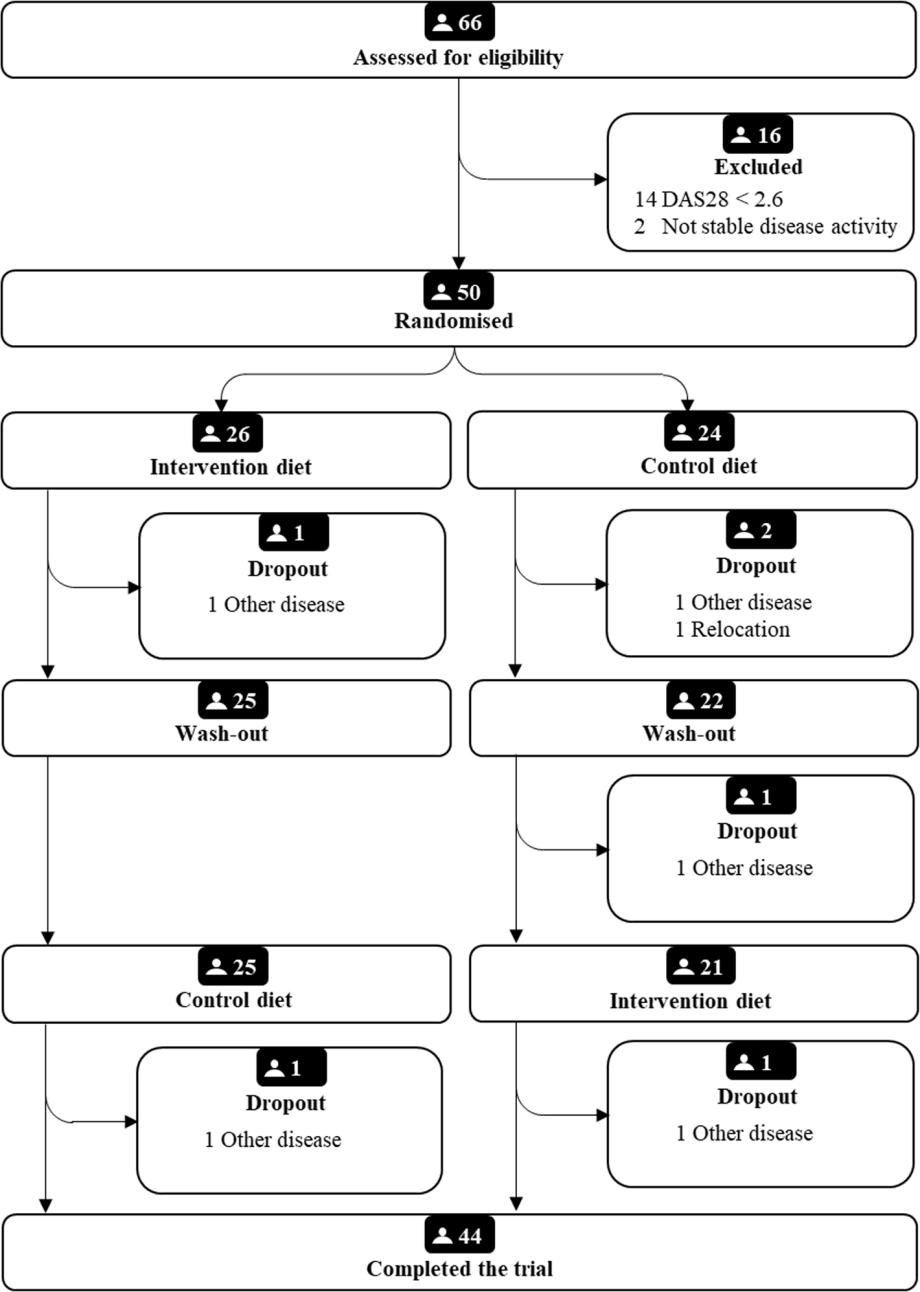
Flow chart of subject recruitment in the ADIRA trial reported according to CONSORT

Out of the 50 participants who entered the trial, 47 completed at least one dietary period and 44 completed both periods (Fig. 1). The intervention period lasted for ten weeks (median 9.6, range 7.9–12.6 weeks), with a 4-month washout period in between (median 16.9, range 9.1–19.9 weeks). Most participants were women of European origin with a university educational level (Table 2). DAS28-ESR indicated moderate disease activity among 57% of participants (Table 3). A majority of participants used at least one conventional synthetic DMARD, and over a third a biologic DMARD (Table 2). Approximately one third of the participants were overweight and one third obese. Central obesity (waist > 80 cm for women and > 94 cm for men) was seen in 70% of males and 75% of females (Table 3). Participants were instructed to keep their weight and there were no significant changes in weight during or between dietary periods. About half of participants participated in physical exercise at least once weekly (Table 2). Seventeen percent of participants had elevated fasting TG-concentrations (> 1.7 mmol/L) at baseline (Table 3), according to European guidelines [4, 15], and half of the participants were taking at least one cardiovascular agent (Table 2). During the trial, adverse events in the form of upset stomach occurred; 13 participants reported gastrointestinal discomfort during intervention period, and four during control period. Overall, compliance to the dietary interventions was high; 96% of participants were compliant during intervention period and 87% during control period.

**Table 2:** Baseline data of participants who completed at least one dietary period reported by sequence

	Intervention-Control ***n*** = 25 n (%)	Control-Intervention ***n*** = 22 n (%)	All n = 47 n (%)
**Demographics**			
Female	20 (80)	16 (73)	36 (77)
Smokers	2 (8)	0 (0)	2 (4.3)
**Origin of parents**			
Europe	23 (92)	21 (95)	44 (94)
Africa	1 (4)	0 (0)	1 (2)
Asia	1 (4)	1 (5)	2 (4)
**Education**			
Junior high school	4 (16)	4 (18)	8 (17)
2 year senior high school	4 (16)	5 (23)	9 (19)
≥ 3 year senior high school	3 (12)	4 (18)	7 (15)
College or university	14 (56)	9 (41)	23 (49)
**Everyday physical activity**			
Sedentary or standing	5 (20)	1 (4.5)	6 (13)
Light but partly physically active	12 (48)	4 (18)	16 (34)
Light and physically active	6 (24)	9 (41)	15 (32)
Sometimes physically straining	2 (8.0)	8 (36)	10 (21)
**Training in exercise outfit, past 3-months**			
Never	2 (8.0)	5 (23)	7 (15)
Every now and then – not regularly	8 (32)	6 (27)	14 (30)
1–2 times/week	7 (28)	3 (14)	10 (21)
2–3 times/week	5 (20)	4 (18)	9 (19)
> 3 times/week	3 (12)	4 (18)	7 (15)
**Medications used at baseline**			
Cardiovascular agents	14 (56)	9 (41)	23 (49)
Vasodilator	9 (36)	8 (36)	17 (36)
Statins	5 (20)	3 (14)	8 (17)
Betablocker	5 (20)	3 (14)	8 (17)
Anticoagulants	1 (4)	3 (14)	4 (9)
Diuretics	3 (12)	2 (9)	5 (11)
bDMARD	8 (32)	10 (45)	18 (38)
csDMARD	19 (76)	16 (73)	35 (74)

*bDMARD* Biological disease-modifying antirheumatic drugs, *csDMARD* Conventional synthetic disease-modifying antirheumatic drugs

**Table 3:** Baseline measurements of participants who completed at least one dietary period reported by diet sequence

	Intervention-Control n = 25 Median (IQR)	Control-Intervention n = 22 Median (IQR)	All n = 47 Median (IQR)
Age (years)	62.8 (59.3, 72.4)	64.3 (47.8, 72.4)	62.8 (53.9, 70.8)
Weight (kg)	75.5 (65.6, 83.4)	78 (70.6, 83.4)	77.8 (66.9, 85.4)
BMI (kg/m^2^)	26.9 (23.5, 30.8)	25.9 (24.1, 30.8)	26.6 (24, 31.8)
Waist (cm)	92 (81.5, 98.3)	92.5 (83.8, 98.3)	92 (83, 100)
Hip (cm)	107 (98.5, 109.5)	105.5 (96.8, 109.5)	106 (98, 112)
SBP (mmHg)	130 (121, 146.5)	140 (126.5, 146.5)	135 (125, 140)
DBP (mmHg)	80 (74.5, 89)	80 (70, 89)	80 (72, 89)
DAS28-ESR	3.8 (3.1, 4.6)	3.3 (2.9, 4.6)	3.7 (3, 4.6)
ESR (mm)	20 (11.5, 25.3)	19.5 (8.8, 25.3)	20 (11, 26)
CRP (mg/L)	3 (1, 6)	2.5 (1, 6)	3 (1, 6)
LDL cholesterol (mmol/L)	3.4 (2.6, 4.3)	3.3 (3, 4.3)	3.3 (2.8, 4.2)
HDL cholesterol (mmol/L)	1.7 (1.5, 2.2)	1.5 (1.3, 2.2)	1.6 (1.3, 2)
Triglycerides (mmol/L)	1.1 (1, 1.6)	1.1 (0.7, 1.6)	1.1 (0.8, 1.6)
Total cholesterol (mmol/L)	5.4 (4.6, 6)	5.6 (4.6, 6)	5.5 (4.6, 6)
Apo-B100 (mg/dL)^a^	82.6 (69.7, 90.2)	82.8 (67.7, 104)	82.7 (69.7, 94.7)
Apo-A1 (mg/dL)^a^	141 (135, 156)	139 (121, 162)	140 (124, 157)
Apo-B100/Apo-A1^a^	0.57 (0.47, 0.65)	0.59 (0.50, 0.76)	0.57 (0.49, 0.71)

Values presented as median (Interquartile range; IQR). *BMI* Body Mass Index, *SBP* Systolic Blood Pressure, *DBP* Diastolic Blood Pressure, *DAS28-ESR* Disease Activity Score Erythrocyte Sedimentation rate, *ESR* Erythrocyte Sedimentation Rate, *LDL* Low Density Lipoprotein, *HDL* High Density Lipoprotein, *Apo-B100* Apolipoprotein B100, *Apo-A1* Apolipoprotein A1

^a^Intervention-control, n = 23; Control-intervention, n = 21; All, n = 44

The fatty acid profile in plasma phospholipids reflected the dietary intake and confirmed compliance at a group level (Table 5). The intervention period resulted in a decrease in percentage of monounsaturated fatty acids (mean − 1.310, 95% CI -1.888, − 0.732) and an increase in polyunsaturated fatty acids (mean 1.611, 95% CI 0.856, 2.366). In contrast, the control period resulted in an increase in saturated fatty acids (mean 0.557, 95% CI 0.070, 1.043) and a decrease in polyunsaturated fatty acids (mean − 1.153 95% CI -1.897, − 0.409).

These changes resulted in significantly different concentrations of saturated, monounsaturated, and polyunsaturated fatty acids between the two dietary periods. Specifically, between-dietary treatment concentrations of eicosapentaenoic acid and docosahexaenoic acid, reflecting fish intake, were significantly higher after the intervention diet compared to the control diet (Table 5).

### Effects of diet on cardiovascular risk factors

There was a significant difference between dietary treatment in TG concentration in favor of the intervention diet (Table 4) (mean: − 0.192 mmol/L; 95% CI -0.328, − 0.057) (*p* = 0.007). Eighty-seven percent of participants reached recommended TG concentrations after the intervention diet, compared to 72% after the control diet (Table 4). Further, there was an increase in HDL-cholesterol in favor of the intervention diet compared to control (mean: 0.074 mmol/L, 95% CI: 0.000, 0.148) (*p* = 0.049) and a decrease in non-HDL-cholesterol (mean: − 0.187 mmol/L, 95% CI:-0.373, − 0.001) (p = 0.049) (Table 4) .

**Table 4:** Modelled estimates of differences in blood lipids and blood pressure between intervention and control^a^

	Intervention (***n*** = 45) Mean Change (95% CIs)	Control (***n*** = 46) Mean Change (95% CIs)	Difference between dietary periods^**b**^	95% CIs	P
Total Cholesterol (mmol/L) ^c^	−0.015 (− 0.187, 0.157)	0.096 (− 0.073, 0.265)	− 0.111	− 0.315, 0.093	0.279
LDL Cholesterol (mmol/L) ^c^	− 0.074 (− 0.220, 0.072)	0.079 (− 0.065, 0.223)	− 0.153	−0.322, 0.016	0.075
HDL Cholesterol (mmol/L) ^c^	0.047 (− 0.033, 0.128)	− 0.027 (− 0.107, 0.053)	0.074	0.000, 0.148	0.049
Triglycerides (mmol/L) ^c^	−0.114 (− 0.211, − 0.018)	0.078 (− 0.017, 0.173)	− 0.192	− 0.328, − 0.057	0.007
Non-HDL Cholesterol (mmol/L) ^c^	− 0.063 (− 0.211, 0.085)	0.124 (− 0.023, 0.270)	− 0.187	− 0.373, − 0.001	0.049
SBP (mmHg) ^d^	− 1.709 (−5.824, 2.407)	− 2.611 (−6.669, 1.447)	0.902	−3.915, 5.720	0.707
DBP (mmHg) ^d^	−2.154 (−4.282, − 0.027)	−4.041 (− 6.134, − 1.948)	1.886	−1.098, 4.871	0.212

^a^Participants completing at least one dietary period (*n* = 47). *LDL* Low Density Lipoprotein, *HDL* High Density Lipoprotein, *SBP* Systolic Blood Pressure, *DBP* Diastolic Blood Pressure

^b^Intervention – Control, post period values

^c^Analysed by use of a linear mixed model with period, treatment, sequence and baseline value as fixed effects and subject as random effect adjusted for sex

^d^Analysed by use of a linear mixed model with period, treatment, sequence and baseline value as fixed effects and subject as random effect

**Table 5:** Modelled estimates of differences in percentage of plasma phospholipid fatty acids between intervention and control^a^

	Intervention (n = 45) Mean Change (95% CIs)	Control (n = 46) Mean Change (95% CIs)	Difference between dietary periods^b^	95% CIs	P
C14:0^c^	− 0.022 (− 0.115, 0.071)	0.156 (0.064, 0.248)	− 0.178	− 0.302, − 0.055	0.006
C16:0^c^	− 0.333 (− 0.702, 0.037)	0.462 (0.099, 0.826)	− 0.795	− 1.255, − 0.335	0.001
C18:0^c^	0.068 (− 0.092, 0.227)	− 0.051 (− 0.208, 0.106)	0.119	− 0.106, 0.343	0.296
C20:0^c,d^	− 0.001 (− 0.006, 0.004)	0.001 (− 0.004, 0.006)	− 0.002	− 0.009, 0.005	0.598
C22:0^c^	0.001 (− 0.002, 0.004)	− 0.001 (− 0.004, 0.001)	0.002	−0.001, 0.005	0.158
C24:0^c^	−0.011 (− 0.017, − 0.005)	0.007 (0.001, 0.013)	−0.018	− 0.026, − 0.010	< 0.001
Σ Saturated fat^c^	− 0.288 (− 0.782, 0.206)	0.557 (0.070, 1.043)	−0.845	− 1.463, − 0.227	0.009
C16:1 n-7^c^	− 0.194 (− 0.303, − 0.085)	0.163 (0.056, 0.271)	−0.358	− 0.511, − 0.205	< 0.001
C18:1 n-9^c^	−1.045 (− 1.538, − 0.551)	0.446 (− 0.040, 0.932)	− 1490	−2.181, − 0.799	< 0.001
C18:1 n-7^c^	−0.067 (− 0.122, − 0.011)	−0.036 (− 0.091, 0.018)	−0.030	− 0.101, 0.041	0.392
C20:1 n-9^c^	−0.005 (− 0.017, 0.008)	−0.009 (− 0.021, 0.004)	0.004	−0.012, 0.020	0.631
C24:1 n-9^c^	0.003 (−0.001, 0.007)	0.001 (−0.003, 0.005)	0.002	−0.003, 0.006	0.408
Σ monounsaturated fat^c^	− 1.310 (− 1.888, − 0.732)	0.561 (− 0.009, 1.130)	−1.870	−2.692, − 1.049	< 0.001
C18:2 n-6^c^	1.520 (0.806, 2.234)	− 0.711 (− 1.414, − 0.007)	2.231	1.219, 3.243	< 0.001
C18:3 n-6^c^	− 0.015 (− 0.042, 0.012)	0.031 (0.005, 0.058)	−0.046	− 0.079, − 0.013	0.007
C20:2 n-6^c^	0.005 (− 0.006, 0.017)	− 0.003 (− 0.015, 0.008)	0.008	0.000, 0.017	0.057
C20:3 n-6^c^	0.143 (0.076, 0.210)	−0.042 (− 0.110, 0.026)	−0.185	− 0.274, − 0.095	< 0.001
C20:4 n-6^c^	−0.484 (− 0.675, − 0.292)	−0.177 (− 0.366, 0.011)	−0.306	− 0.575, − 0.038	0.026
Σ n-6 fat^c^	0.989 (0.228, 1.750)	−0.719 (− 1.469, 0.030)	1.708	0.622, 2.794	0.003
C18:3 n-3^c^	−0.003 (− 0.056, 0.050)	−0.019 (− 0.071, 0.033)	0.016	−0.049, 0.080	0.623
C20:4 n-3^c^	0.005 (− 0.010, 0.020)	0.022 (0.007, 0.036)	−0.017	− 0.035, 0.001	0.064
C20:5 n-3^c^	0.212 (0.053, 0.370)	−0.180 (− 0.336, − 0.024)	0.391	0.204, 0.579	< 0.001
C22:5 n-3^c^	0.000 (− 0.020, 0.019)	0.013 (− 0.007, 0.032)	− 0.013	−0.040, 0.014	0.327
C22:6 n-3^c^	0.406 (0.273, 0.540)	−0.272 (− 0.403, − 0.141)	0.678	0.491, 0.865	< 0.001
Σ n-3 fat^c^	0.620 (0.318, 0.921)	−0.436 (− 0.733, − 0.139)	1.056	0.674, 1.438	< 0.001
Σ polyunsaturated fat^c^	1.611 (0.856, 2.366)	−1.153 (− 1.897, − 0.409)	2.765	1.695, 3.835	< 0.001

^a^Participants completing at least one dietary period (*n* = 47)

^b^Intervention – Control, post period values

^c^Analysed by use of a linear mixed model with period, treatment, sequence and baseline value as fixed effects and subject as random effect

^d^Adjusted for BMI

**Table 6:** Modelled estimates of differences in apolipoprotein, lipoproteins and lipids between intervention and control^a^

	Intervention (***n*** = 42) Mean Change (95% CIs)	Control (***n*** = 43) Mean Change (95% CIs)	Difference between dietary periods^b^	95% CIs	P
**Apolipoprotein concentrations (mg/dL)**					
Apo-A1	2.782 (− 1.491, 7.054)	1.111 (− 3.104, 5.327)	1.670	− 3.505, 6.846	0.517
Apo-B100	− 0.325 (− 3.758, 3.108)	4.130 (0.741, 7.520)	− 4.455	−8.331, − 0.578	0.026
Apo-B100/Apo-A1	− 0.016 (− 0.039, 0.007)	0.022 (0.000, 0.045)	− 0.038	− 0.066, − 0.011	0.007
**Particle numbers (nmol/L)**					
VLDL	−5.750 (− 15.270, 3.771)	4.835 (− 4.535, 14.205)	− 10.585	− 23.951, 2.782	0.119
IDL	−2.813 (− 9.383, 3.757)	7.429 (0.958, 13.899)	− 10.241	− 18.699, − 1.783	0.019
LDL	7.210 (− 53.413, 67.833)	62.424 (2.563, 122.285)	−55.214	−123.396, 12.968	0.109
**Triglycerides in lipoprotein particles (mg/dL)**					
VLDL	−11.762 (− 19.999, −3.526)	5.072 (− 3.038, 13.182)	− 16.834	−28.404, − 5.264	0.005
IDL	−2.022 (− 3.448, − 0.595)	1.068 (− 0.337, 2.473)	−3.090	−5.092, − 1.088	0.003
LDL	0.054 (− 0.840, 0.949)	0.870 (− 0.011, 1.750)	− 0.815	− 1.976, 0.345	0.163
HDL	−0.171 (− 0.726, 0.385)	0.586 (0.039, 1.133)	− 0.757	− 1.530, 0.016	0.055
**Cholesterol in HDL particles (mg/dL)**					
HDL-1	2.461 (0.922, 4.001)	−0.625 (− 2.145, 0.894)	3.087	1.303, 4.871	0.001
HDL-2	0.327 (−0.086, 0.741)	−0.181 (− 0.590, 0.227)	0.509	0.063, 0.955	0.027
HDL-3	0.045 (−0.339, 0.430)	−0.012 (− 0.390, 0.367)	0.057	−0.459, 0.573	0.824
HDL-4	−0.223 (− 0.976, 0.530)	0.282 (− 0.459, 1.023)	−0.505	−1.524, 0.513	0.320

^a^Participants completing at least one dietary period and with coherent results between clinical data and NMR-analysis (*n* = 44), analyzed by use of a linear mixed model with period, treatment, sequence and baseline value as fixed effects and subject as random effect adjusted for sex. Apo-A1, Apolipoprotein A1; Apo-B100, Apolipoprotein B100; VLDL, Very low density lipoprotein; IDL, Intermediate density lipoprotein; LDL, low density lipoprotein, HDL, High density lipoprotein; HDL-1, 1.063–1.100 kg/L; HDL-2, 1.100–1.112 kg/L; HDL-3, 1.112–1.125 kg/L; HDL-4, 1.125–1.210 kg/L

^b^Intervention – Control, post period values

The Apo-B100/Apo-A1 ratio indicated a shift towards a less atherogenic lipoprotein profile in favor of the intervention compared to the control (Table 6) (mean: -0.038; 95% CI -0.066, − 0.011) (p = 0.007). In accordance with this, intermediate density lipoprotein (IDL) particle numbers increased during the control period yielding a significant difference between diet treatment in favor of intervention (mean − 10.241 nmol/L; 95% CI -18.669, − 1.783) (*p* = 0.019) (Table 6). There was a decrease in very low density lipoprotein (VLDL)- and IDL-TG concentrations in favor of the intervention, with a trend also in HDL-TG, but not in LDL-TG (Table 6). Between diet treatment, change was also seen in the lower density (range 1.063–1.112 kg/L) HDL particles in favor of intervention diet (Table 6).

### Interaction analysis

Interaction analysis revealed that SCORE 2015, an estimate of the 10-year CVD mortality, at baseline affected changes in HDL-cholesterol. Participants with low SCORE 2015 (*n* = 27) exhibited a significant (*p* = 0.022) between-dietary treatment effect in favor of the intervention (mean 0.117 mmol/L, 95% CI: 0.019, 0.215), whereas those with high SCORE 2015 (*n* = 20) did not experience any significant changes.

Several baseline characteristics affected changes in TG concentrations. Overweight subjects (*n* = 32) showed a decrease in TG during the intervention (mean: − 0.204 mmol/L, 95% CI: − 0.330, − 0.077) and a significant between-dietary treatment effect (mean: − 0.299 mmol/L, 95% CI -0.471, − 0.128) (*p* = 0.001). In contrast, normal weight participants (*n* = 19) showed no such effects. Further, participants with a low baseline fiber intake (*n* = 26) had a significant (*p* = 0.008) between-dietary treatment effect in favor of the intervention on TG concentrations (mean: − 0.257 mmol/L, 95% CI: − 0.437, − 0.076), whereas those with a high baseline dietary fiber intake (*n* = 21) showed no such change. Similarly, those with low baseline dietary quality (*n* = 24) showed significant decrease in TG during the intervention (mean: − 0.183 mmol/L, 95% CI: − 0.338, − 0.028) and showed a significant difference between dietary treatment (mean: − 0.322 mmol/L, 95% CI: − 0.540, − 0.104) (*p* = 0.006). In contrast, those with an overall high dietary quality (*n* = 23) did not significantly alter TG-concentrations during or between any dietary period.

LDL-cholesterol changes were affected by baseline fiber intake; those with low habitual fiber intake (n = 26) had no changes, whereas those with high fiber intake (n = 21) had significant between-dietary changes in favor of intervention (mean: − 0.342 mmol/L, 95% CI: − 0.556, − 0.128) (*p* = 0.003).

### Sensitivity analysis

The sensitivity analysis, including only participants who completed both dietary periods, had high compliance and without new or discontinued DMARD, glucocorticoid or statin treatment (*n* = 28), yielded similar results as the main analysis although fewer results reached significance (Supplemental Table 1).

## Discussion

This randomized controlled cross-over study aimed to test whether a proposed anti-inflammatory diet rich in whole grain, fatty fish, nuts, vegetables and fruit, and supplemented with probiotics, and thus potentially cardio-protective, could improve the risk profile for CVD during stable disease activity in patients with RA, compared to a diet similar to the average dietary intake in Sweden.

The results demonstrate improvement in blood lipid profile and Apo-B100/Apo-A1 ratio, which are important markers of future CVD risk. Plasma phospholipid fatty acid profile, an objective marker of compliance at a group level, reflected the diets and was significantly improved after the intervention dietary period compared to control dietary period. Self-reported compliance was high, and a sensitivity analysis excluding those with lower self-reported compliance and medication changes did not alter any conclusion.

To date, few intervention studies exist on patients with RA using the whole diet approach. The ADIRA intervention diet is a type of healthy Nordic diet that resembles the Mediterranean diet. A TG-lowering effect from the marine n-3 fatty acids EPA and DHA is well described in the literature, while effects from increased fish intake are not equally well documented [16]. Here, we demonstrate a considerable TG-lowering effect from a realistic and palatable whole diet rich in fish. In contrast to our findings, Sköldstam et al. reported significant weight loss and a concurrent decrease in C-reactive protein from a Mediterranean diet intervention, but no between group effects on total cholesterol or TG concentrations [17]. In comparison to the trial by Sköldstam el al., the difference in n-3 fatty acid content between diets in the current study was slightly higher [11, 18], and our cross-over design could lead to higher precision in detecting changes.

Similarly, McKellar et al. also found no effect on TG or total cholesterol concentration in a trial combining Mediterranean diet with marine foods, through instructions and cooking classes in women with RA living in areas of social deprivation [19]. A comparative strength of the ADIRA trial is that foods were home-delivered free of charge to participants, which could have led to higher compliance.

The observed TG-lowering effect was most prominent in the lipoproteins responsible for TG-transport in the fasting state; VLDL and IDL particles (Table 6). In line with this, IDL which are formed from VLDL in the circulation, decreased in particle numbers, suggesting the intervention, rich in n-3 fatty acids and low in refined carbohydrates, might have led to decreased VLDL synthesis.

HDL-cholesterol tends to decrease with raised inflammatory activity and seems to be lower in patients with RA compared to healthy controls [20]. In our trial, total HDL-cholesterol content increased and specifically so in some HDL-subtypes. Interpretations from HDL-subtype analysis are however limited due to varying methods of analysis, which complicates comparisons between the literature and the data in our trial. The Apo-B100/Apo-A1 ratio however, the most significant overall CVD risk marker [21], improved significantly in favor of the intervention diet in our trial.

Due to increased clearance in a state of elevated inflammation, blood cholesterol concentrations inversely follow changes in biomarkers of inflammation, and normalize upon resolved inflammation [6]. In our trial, there were no indications of increased inflammation during the intervention period compared to control period. It is possible that the observed increases in HDL-cholesterol in the intervention diet stem from lowered inflammation. However, non-HDL-cholesterol decreased significantly in favor of intervention, indicating that the changes in blood cholesterol cannot be attributed to reduced inflammation alone. Changes in plasma fatty acid composition further indicates a plausible biological mechanism behind the lowered TG-concentrations, independent of changes in inflammation. Thus, we believe the improved lipid profile likely translates to a CVD risk reduction beyond the potential effects on systemic inflammation.

In the ADIRA trial, several characteristics affected the response to the intervention: overweight participants and those with poor overall dietary quality exhibited the strongest reduction in TG concentration. Likewise, those with low habitual fiber intake showed a more pronounced decrease in TG concentrations between dietary periods. Although no significant effect was seen on LDL-cholesterol in our main analysis, a difference between dietary periods was seen among those with a high habitual fiber intake, driven by an increase during the control period, suggesting habitual intake was better than control diet in these individuals.

HDL-cholesterol increased during the intervention dietary period in participants with low SCORE 2015. There is no clear explanation for this finding. The scoring method is however imprecise, and it is possible that responders clustered by chance.

The current trial has several strengths. We made an effort to control for inter-individual variation by using a cross-over design, which is important for a group such as patients with RA where disease activity and medication exert effects on metabolism. We used the Swedish Rheumatology Quality Register to invite all eligible patients in the area where food delivery was possible, thus minimizing selection bias. Only patients with active and stable disease activity, measured by DAS28-ESR, were included. Participants continued habitual pharmacological treatment, making the results more representative to the patient population as a whole. Use of medication was self-reported at screening and information on any changes during the dietary periods was recorded. Efforts were made to blind participants to the intervention. We collected dietary data during both dietary periods in order to characterize the dietary intake as a whole, and measured compliance with self-reported intake data as well as with plasma fatty acids at the group level. Furthermore, staff instructed the participants to keep a stable weight and no significant weight changes between dietary periods were detected; hence, the effects shown on blood lipid profile are unlikely to stem from weight change, but are likely to reflect the dietary interventions.

There are also some limitations to the study. Sample size was based on a power calculation aimed at detecting relevant changes in DAS28-ESR, not blood lipid concentrations. It is thus possible that we could have seen more clear effects had we recruited specifically to detect changes in blood lipids. This report explores the moderating effects from participant characteristics in an interaction analysis. While this yields valuable information about determinants of response, not adjusting our analyses for multiplicity testing increases the risk of type 1 errors. *P*-values therefore need to be interpreted with caution. We have also not been able to estimate lipoprotein particle function, such as, for example, HDL-efflux capacity. Further, our study population was rather heterogeneous in terms of BMI distribution; obesity could alter the response to diet. The participants in our trial had a high educational level and high reported compliance; this might not always be the case and could thus limit the generalization of our results to other population groups. About half of the participants were medicated with the aim to reduce cardiovascular risk factors. This represents the clinical reality and increases external validity, but may have limited the effect size of our dietary intervention.

Other Mediterranean diet interventions have shown promising effects on CVD-prevention [8]. The largest RCT to date (*n* = 7447), found a 30% reduced hazard ratio for CVD events after 4.8 years of intervention with Mediterranean diet [22]. A subset (*n* = 772) of this study population was examined already after 3 month into the intervention, with comparable effects on TG and HDL-cholesterol as those presented in the ADIRA trial [23]. The long term effects of these findings in patients with RA are unknown, but the results of the current study suggest that dietary treatment should be included in cardiovascular risk management for this patient group.

## Conclusion

Blood lipid profile, plasma fatty acid composition and Apo-B100/Apo-A1 ratio were significantly improved by a proposed anti-inflammatory portfolio diet, indicating that adjuvant dietary treatment can be beneficial for CVD prevention in RA, even among pharmacologically well-managed patients.

## Supplementary Information

**Additional file 1 Supplemental Table 1.** Modelled estimates of differences in blood lipids between intervention and control in a sensitivity analysis.

## Data Availability

The datasets during and/or analysed during the current study available from the corresponding author on reasonable request. Swedish law forbids data to be shared publicly.

## Abbreviations

Apo-A1: Apolipoprotein A1
Apo-B100: Apolipoprotein B100
bDMARD: biologic disease-modifying antirheumatic drugs
BMI: Body mass index
csDMARD: conventional synthetic disease-modifying antirheumatic drugs
CVD: Cardiovascular disease
DAS28-ESR: 28-joint Disease Activity Score Erythrocyte Sedimentation Rate
DBP: Diastolic blood pressure
DMARD: disease-modifying antirheumatic drugs
HDL: High density lipoprotein
IDL: Intermediate density lipoprotein
IVDr: In Vitro Diagnostics Research
LDL: Low density lipoprotein
NMR: Nuclear Magnetic Resonance
RA: Rheumatoid arthritis
SBP: Systolic blood pressure
Score 2015: Systematic Coronary Risk Evaluation 2015
TG: Triglycerides
VLDL: Very low density lipoprotein
