# Supplementary material for: Diet intervention improves cardiovascular profile in patients with rheumatoid arthritis: results from the randomized controlled cross-over trial ADIRA
Source: Nutr J. 2021 Jan 23;20:9. doi: 10.1186/s12937-021-00663-y (PMC7827982; doi:10.1186/s12937-021-00663-y)
Supplement: Supplementary file 1 — Additional file 1 Supplemental Table 1. Modelled estimates of differences in blood lipids between intervention and control in a sensitivity analysis. [file 12937_2021_663_MOESM1_ESM.docx]

Supplemental table 1. Modelled estimates of differences in blood lipids between intervention and control in a sensitivity analysis^1^

|  | **Intervention**  **(n=28)**  **Mean Change (95% CIs)** | **Control**  **(n=28)**  **Mean Change (95% CIs)** | **Difference**  **between dietary periods^2^** | **95% CIs** | **P** |
| --- | --- | --- | --- | --- | --- |
| Total Cholesterol  (mmol/L)^3^ | -0.049 (-0.237, 0.139) | 0.021 (-0.167, 0.209) | -0.070 | -0.312, 0.172 | 0.557 |
| LDL Cholesterol  (mmol/L)^3^ | -0.138 (-0.288, 0.013) | 0.008 (-0.143, 0.158) | -0.146 | -0.353, 0.062 | 0.161 |
| HDL Cholesterol  (mmol/L)^3^ | 0.054 (-0.064, 0.172) | -0.049 (-0.167, 0.069) | 0.103 | 0.008, 0.197 | 0.034 |
| Triglycerides  (mmol/L)^3^ | -0.081 (-0.216, 0.054) | 0.086 (-0.050, 0.221) | -0.167 | -0.343, 0.010 | 0.063 |
| Non-HDL Cholesterol  (mmol/L)^3^ | -0.106 (-0.252, 0.040) | 0.075 (-0.071, 0.220) | -0.181 | -0.387, 0.026 | 0.085 |

^1^Participants completing the entire trial, who self-reported an intake of ≥ 80% of the supplied meals, with no new or discontinued DMARD, glucocorticoid or statin treatment (n=28). LDL, Low Density Lipoprotein; HDL, High Density Lipoprotein.

^2^Intervention – Control, post period values.

^3^Analyzed by use of a linear mixed model with period, treatment, sequence and baseline value as fixed effects and subject as random effect adjusted for sex.
